# Association between PM_2.5_-bound metals and pediatric respiratory health in Guangzhou: An ecological study investigating source, health risk, and effect

**DOI:** 10.3389/fpubh.2023.1137933

**Published:** 2023-03-09

**Authors:** Yi Zheng, Sili Chen, Yuyang Chen, Jingye Li, Binhe Xu, Tongxing Shi, Qiaoyuan Yang

**Affiliations:** ^1^Department of Preventive Medicine, School of Public Health, Guangzhou Medical University, Guangzhou, China; ^2^Department of Anesthesiology, School of Anesthesiology, Southern Medical University, Guangzhou, China; ^3^Department of Clinical Medicine, Basic Medicine College, Zunyi Medical University, Zunyi, China; ^4^Department of Environmental Health, Guangzhou Center for Disease Control and Prevention, Guangzhou, China; ^5^Department of Environmental Health, Institute of Public Health, Guangzhou Medical University, Guangzhou, China

**Keywords:** PM_2.5_-bound metals, source identification, children, health risk, time-series study

## Abstract

**Background:**

The adverse effects of 2.5-μm particulate matter (PM_2.5_) exposure on public health have become an increasing concern worldwide. However, epidemiological findings on the effects of PM_2.5_-bound metals on children's respiratory health are limited and inconsistent because PM_2.5_ is a complicated mixture.

**Objectives:**

Given the vulnerability of children's respiratory system, aim to pediatric respiratory health, this study evaluated the potential sources, health risks, and acute health effects of ambient PM_2.5_-bound metals among children in Guangzhou, China from January 2017 to December 2019.

**Methods:**

Potential sources of PM_2.5_-bound metals were detected using positive matrix factorization (PMF). A health risk assessment was conducted to investigate the inhalation risk of PM_2.5_-bound metals in children. The associations between PM_2.5_-bound metals and pediatric respiratory outpatient visits were examined with a quasi-Poisson generalized additive model (GAM).

**Results:**

During 2017–2019, the daily mean concentrations of PM_2.5_ was 53.39 μg/m^3^, and the daily mean concentrations of PM_2.5_-bound metals range 0.03 ng/m^3^ [thorium (Th) and beryllium (Be)] from to 396.40 ng/m^3^ [iron (Fe)]. PM_2.5_-bound metals were mainly contributed by motor vehicles and street dust. PM_2.5_-bound arsenic (As), cadmium (Cd), cobalt (Co), chromium (Cr)(VI), nickel (Ni), and lead (Pb) were found to pose a carcinogenic risk (CR). A quasi-Poisson GAM was constructed that showed there were significant associations between PM_2.5_ concentrations and pediatric outpatient visits for respiratory diseases. PM_2.5_ was significantly associated with pediatric outpatient visits for respiratory diseases. Moreover, with a 10 μg/m^3^ increase in Ni, Cr(VI), Ni, and As concentrations, the corresponding pediatric outpatient visits for respiratory diseases increased by 2.89% (95% *CI*: 2.28–3.50%), acute upper respiratory infections (AURIs) increased by 2.74% (2.13–3.35%), influenza and pneumonia (FLU&PN) increased by 23.36% (20.09–26.72%), and acute lower respiratory infections (ALRIs) increased by 16.86% (15.16–18.60%), respectively.

**Conclusions:**

Our findings showed that PM_2.5_ and PM_2.5_-bound As, Cd, Co, Cr(VI), Ni, and Pb had adverse effects on pediatric respiratory health during the study period. New strategies are required to decrease the production of PM_2.5_ and PM_2.5_-bound metals by motor vehicles and to reduce levels of street dust to reduce children's exposure to these pollutants and thereby increase child health.

## Introduction

The International Agency for Research on Cancer (IARC) defines outdoor particulate matter (PM) as a Group 1 carcinogen in humans (1). Exposure to fine particulate matter (PM_2.5_; particulate matter with an aerodynamic diameter of ≤2.5 μm) has significantly increased the burden of disease globally, mainly by increasing the risk of diabetes, cardiovascular diseases, poor respiratory health, and lung cancer (2, 3). In China, PM_2.5_ exposure is one of the top four risk factors for death from stroke, ischemic heart disease, chronic obstructive pulmonary disease (COPD), and lung cancer (2).

The small size and large specific surface area of PM_2.5_ means that it can easily bind toxic compounds, including metals. Among these, PM_2.5_-bound metals are key contributors to PM_2.5_ toxicity and can cause severe adverse health effects. PM_2.5_-bound metals can enter the body by ingestion, dermal contact, or by inhalation, which allows them to affect cardiopulmonary function and distribute to organs through the blood circulation (4). Several epidemiological studies have found that exposure to PM_2.5_-bound nickel (Ni), vanadium (V), lead (Pb), and zinc (Zn) can increase cardiopulmonary diseases, and that exposure to PM_2.5_-bound arsenic (As), cobalt (Co), Ni, manganese (Mn), and chromium (Cr) can trigger the production of reactive oxygen species and thereby cause respiratory inflammation (5–9). However, owing to on the inconsistency of PM_2.5_-bound metals, there is a limited epidemiological understanding of their effects on respiratory health (10–13). Therefore, it is critical to investigate the health effects of PM_2.5_-bound metals further.

A better understanding of the sources of PM_2.5_-bound metals and the identification of those metals that are most harmful to children's health would assist decision-makers to develop air pollution regulations and provide directions for basic research. Positive matrix factorization (PMF) is an analytical method that has been widely employed to determine the contributions of various sources of PM_2.5_ to air pollution and is recommended by the United States Environmental Protection Agency (USEPA) (14, 15). Moreover, health risk assessments are recommended by the USEPA and the National Health Commission of the People's Republic of China (16, 17) for identifying heavy metals that pose carcinogenic and non-carcinogenic risks to humans via inhalation (8). A generalized additive model (GAM) with a quasi-Poisson link can be used for time series analysis of the short-term association between ambient air pollution and outpatient visits, as it enables control for long-term trends, day of the week (DOW), temperature, and humidity (18). Thus, we used this model to determine the most significant associations between PM_2.5_-bound metals and respiratory diseases in children.

Guangzhou is the third-largest city in China and is one of the top four first-tier cities in the country. Due to rapid social and economic development, air pollution condition in Guangzhou has become more serious. Present study on the sources of PM_2.5_-bound metals in Guangzhou mainly focused on total PM, and the results show that the main sources are traffic emissions, soil dust, and biomass burning (19). However, since the distribution spectrum of metal is specific to different sources, it is of public health importance to study the sources of PM_2.5_-bound metals and to discover the effects on the population for better regulation of different sources in Guangzhou. In addition, many studies have demonstrated that children are more vulnerable than adults to the adverse effects of air pollution for many reasons. These include the fact that children breathe twice as quickly as adults, children's lungs are still growing, and children spend more time outside than adults (20, 21). Hence, comprehensive studies are needed to determine the characteristics of air pollution and the relationship between metal-containing pollution and respiratory diseases in children.

The major objectives of this study were to (1) use PMF to determine the sources of PM_2.5_ and PM_2.5_-bound metals air pollution and estimate their contribution to overall PM_2.5_ and PM_2.5_-bound metals air pollution (as a ratio); (2) identify the non-carcinogenic and carcinogenic risks (CR) posed by PM_2.5_-bound metals to children; and (3) use a GAM to investigate the short-term relationships between air concentrations of PM_2.5_-bound metals and daily pediatric outpatient visits for respiratory diseases.

## Materials and methods

### Study location

Guangzhou (22°26′-23°56′ N, 112°57′-114°3′ E) is located in the south of China and is the primary economic and cultural city in the Pearl River Delta. It covers an area of 7,434 km^2^ and had a residential population of 8,970,000 in 2017. Due to its location, Guangzhou is an influential port and transportation hub for Guangdong province. Guangzhou has a humid, warm, tropical/subtropical climate, so its weather is hot and humid in summer and mild and dry in winter. The annual average temperature is 22°C, and the annual average humidity is 80%. Yuexiu district is one of the “four old districts” in Guangzhou and from 2017 to 2019 was ranked 10^th^ out of the 11 districts of Guangzhou in terms of air pollution, and Liwan district ranked 11^th^. Yuexiu district also has the largest population of all districts in Guangzhou, with children younger than 18 years accounting for 14.41% of its population.

### Hospital outpatients

Data from January 1, 2017 to December 31, 2019 were obtained from Guangzhou Yuexiu District Children's Hospital, which is one of the two specialized children's hospitals in Guangzhou. During the study period, individuals who visited the respiratory pediatric department of this hospital and were diagnosed with respiratory disease [International Statistical Classification of Diseases and Related Health Problems 10th revision (ICD-10) codes: J00–J99 and R04–R9.3] were identified. Specifically, patients who over 18 years of age would be excluded, and the respiratory diseases would be further divided into three kind of specific diseases: acute upper respiratory infections (AURIs, ICD-10 codes J00–J06), influenza and pneumonia (FLU&PN, J09–J18), and acute lower respiratory infections (ALRIs, J20–J22).

### PM_2.5_-bound metals and meteorological data

We collected 24-h mean concentrations of PM_2.5_ and 26 metals from the air monitoring station of the Guangdong Environment Monitoring Center in Yuexiu district for 265 days. The 26 metals were silver (Ag), aluminum (Al), As, barium (Ba), beryllium (Be), bismuth (Bi), Cd, Co, chromium (Cr), copper (Cu), iron (Fe), mercury (Hg), lithium (Li), Ni, Mn, molybdenum (Mo), Pb, antimony (Sb), selenium (Se), tin (Sn), strontium (Sr), thorium (Th), thallium (Tl), uranium (U), V, and Zn. The samples were obtained approximately in the middle of every month, for an average of approximately 10 days, from 2017 to 2019. The air pollution monitor was located approximately 10–20 m above ground level. It was regularly maintained in accordance with the standard operating procedures described in the China National Quality Control Assurance Plan. We also obtained the daily 24-h mean PM_2.5_ mass from 2017 to 2019 (674 days).

The daily meteorological data of temperature (°C) and relative humidity (%) in the study period were obtained from the Guangdong Meteorological Service.

### Statistical analysis

#### PMF

We used EPA PMF 5.0 to quantify the source contributions of PM_2.5_-bound metals. The number of sources was calculated based on Cattell's scree test. The mathematical expression of PMF can be written as follows (Equation 1):


(1) $$\begin{array}{l} X_{ij}=\overset{p}{\underset{k=1}{\sum }}G_{ik}F_{kj}+E_{ij}\; \end{array}$$

where *X*_*ij*_ is the concentration of metal *j* in sample *i*; *p* is the number of pollution sources; *G*_*ik*_ is the factor contribution of source *p* to sample *i*; *F*_*kj*_ is the factor concentration of pollutant *j* from source *p*; and *E*_*ij*_ is the residual.

*G*_*ik*_ and *F*_*kj*_ can be calculated by minimizing the objective function *Q* (Equation 2), as follows:


(2) $$\begin{array}{l} Q=\overset{n}{\underset{i=1}{\sum }}\overset{m}{\underset{j=1}{\sum }}\left(\frac{X_{ij}-\overset{p}{\underset{k=1}{\sum }}G_{ik}F_{kj}}{U_{ij}}\right)=\overset{n}{\underset{i=1}{\sum }}\overset{m}{\underset{j=1}{\sum }}{\left(\frac{E_{ij}}{U_{ij}}\right)}^{2} \end{array}$$

where *U*_*ij*_ is the uncertainty in the concentration of each PM_2.5_-bound metal component.

Finally, because we did not know the detection limit for the measurement method, we calculated the uncertainty as follows (Equation 3) (22):


(3) $$\begin{array}{l} U_{ij}=\sqrt{{\left(c_{j}\times SD\right)}^{2}+{\left(0.05\times X_{ij}\right)}^{2}} \end{array}$$

where *c*_*j*_ is the relative uncertainty for high values of the measured parameter, and *SD* is the standard deviation of *X*_*ij*_.

#### Health risk assessment

To assess the health risk of inhaled PM_2.5_-bound metals in children, we adopted the model recommended by the standards of both the USEPA and the National Health Commission of the People's Republic of China. According to previous research (8, 23), Al, As, Ba, Cd, Co, Cr(VI), Mn, Ni, Se, and V are considered to be non-carcinogenic, while As, Cd, Co, Cr(VI), Ni, and Pb are considered to be both carcinogenic and non-carcinogenic. Daily respiratory tract exposure concentrations were estimated by Equation (4), as follows:


(4) $$\begin{array}{l} EC=\left(C\times ET\times EF\times ED\;\times ASF\right)÷AT \end{array}$$

where *EC* is the average daily concentration of metal exposure through inhalation (μg/m^3^); *C* is the concentration of metals in PM_2.5_ (μg/m^3^); *ET* is the exposure time (24 h/d for children), *EF* is the exposure time (365 d/year for children), *ED* is the exposure duration (18 years for children); *ASF* is the age sensitivity factor (3 for children); and *AT* is the average contact time (18 *years* × 365 *d*/*year* × 24 *h*/*d* for non-carcinogenic risk and 70 *years* × 365 *d*/*year* × 24 *h*/*d* for carcinogenic risk).

The carcinogenic and non-carcinogenic risks were further estimated based on *EC*. The non-carcinogenic risk level was calculated by Equation (5), as follows:


(5) $$\begin{array}{l} HQ=EC÷\left(RfC\times 1000\right) \end{array}$$

where *HQ* is the hazard quotient, which represents the non-carcinogenic risk for PM_2.5_-bound metals, and the *RfC* is the maximum daily reference concentration (Table 1), above which there will be a non-carcinogenic risk over a child's lifetime (mg/m^3^). *HQ* < 1 indicates that there will be no adverse health effects, while *HQ* > 1 indicates that non-carcinogenic effects are possible.

**Table 1:** Summary of non-carcinogenic and carcinogenic risks from exposure to PM_2.5_-bound metals via inhalation in children.

**Measure**	**Al^a^**	**As^a^**	**Ba^b^**	**Cd^a^**	**Co^a^**	**Cr(VI)^a^**	**Mn^a^**	**Ni^a^**	**Pb^a^**	**Se^a^**	**V^c^**
IUR^e^	–	4.30 × 10^−3^	–	1.80 × 10^−3^	9.00 × 10^−3^	8.40 × 10^−2^	–	2.40 × 10^−4^	1.20 × 10^−5^	–	–
RfC^e^	5.00 × 10^−3^	1.50 × 10^−3^	5.00 × 10^−4^	1.00 × 10^−5^	6.00 × 10^−6^	1.00 × 10^−4^	5.00 × 10^−5^	1.40 × 10^−5^	–	2.00 × 10^−2^	1.00 × 10^−4^
HQ^e^	6.85 × 10^−2^	1.21 × 10^−2^	1.06 × 10^−1^	3.66 × 10^−1^	1.30 × 10^−1^	1.61 × 10^−2^	1.51^*^	5.29 × 10^−1^	–	3.81 × 10^−4^	1.37 × 10^−1^
CR^e^	–	7.80 × 10^−5*^	–	6.59 × 10^−6*^	7.02 × 10^−6*^	1.35 × 10^−4**^	–	1.78 × 10^−6*^	1.16 × 10^−6*^	–	–

a Data from Integrated Risk Information System (IRIS), Health Effects Assessment Summary Tables (HEAST), Agency for Toxic Substances and Disease Registry (ATSDR).

b “–” indicates that no recommended parameters were identified or the parameter did not have units.

c *IUR* units are (μg/m^3^)^−1^, *RfC* units are (mg/m^3^)^−1^.

^d^“^*^” indicates *HQ* > 1 or *CR* between 1 × 10^−6^ and 1 × 10^−4^, “^**^” indicates *CR* > 1 × 10^−4^.

e *IUR* represents the inhalation unit risk, *RfC* represents the maximum daily reference concentration *HQ* represents the hazard quotient, *CR* represents carcinogenic risk.

Carcinogenic risk (*CR*), which represents the risk of developing cancer due to exposure to PM_2.5_-bound metals, was calculated by Equation (6), as follows:

(6) $$\begin{array}{l} CR=IUR\times EC \end{array}$$

where *IUR* is the inhalation unit risk (m^3^/μg). *CR* < 1 × 10^−6^ indicates a negligible carcinogenic risk in children; *CR* = 1 × 10^−6^-1 × 10^−4^ indicates a possible carcinogenic risk in children; and *CR* > 10^−4^ indicates a high carcinogenic risk in children and therefore attention is needed.

#### GAM

We used two-stage Poisson regression in a GAM to examine the associations between pediatric outpatient visits for respiratory diseases and daily PM_2.5_ and PM_2.5_-bound metal concentrations, respectively. This model complies with the recommendations of the National Health Commission of the People's Republic of China (17). Due to missing data—the lack of daily concentration data for PM_2.5_-bound metals—we followed previous studies by computing the ratio of the monthly mean concentration of PM_2.5_-bound metals to the daily PM_2.5_ concentration (13, 24). We controlled for seasonality and long-term trends in the model by using penalized smoothing splines with six degrees of freedom (*df* ) per year. We also included an indicator variable for the DOW and the daily mean temperature and relative humidity, each with three *df*. Consistent with a previous study (25), we calculated the effect of PM_2.5_ exposure with a lag of 0–7 days and a moving average of lag days 01–07, in addition to the effect of PM_2.5_-bound metal exposure with a lag of 0 days.

In the first stage, we fitted a time series analysis of daily pediatric outpatient visits for respiratory disease and daily PM_2.5_ concentration (Equation 7). In the second stage, we used the PM_2.5_ concentration, the ratio between the monthly mean concentration of the PM_2.5_-bound metal and the daily PM_2.5_ concentration, and the number of daily pediatric outpatient visits for respiratory diseases in Equation (8). These two equations are presented below:


(7) $$\begin{array}{r} \begin{array}{r} \log E(Y_{t})=\beta_{0}Z_{t}+s(time,df)+s(X_{t},df)+DOW+\alpha \\ \log E(Y_{t})=\beta_{1}Z_{t}+s(time,df)+s(X_{t},df)+DOW+ \end{array}\\ \begin{array}{r} \alpha +\beta_{2}\;monthly\;metal\;concentration/PM_{2.5}+ \end{array} \end{array}$$

(8) $$\begin{array}{l} \beta_{3}\;monthly\;metal\;concentration/PM_{2.5}\times PM_{2.5} \end{array}$$

where *E(Y*_*t*_*)* is the expected number of daily pediatric outpatient visits for respiratory diseases on day *t*; β_0_–β_3_ are the regression coefficients, where β_0_–β_2_ represent the main effects of PM_2.5_ and the ratio of the monthly mean concentration of PM_2.5_-bound metals to the daily PM_2.5_ concentration and β_3_ is the interaction term; *Z*_*t*_ is the daily PM_2.5_ concentration; *s* is the penalized smoothing spline function; *df* are the degrees of freedom; *X*_*t*_ is the meteorological factor, which includes daily mean temperature and relative humidity, *DOW* is the day of week, which was used to adjust for the DOW effect; and *a* is the intercept. We reported the results as the percent change in excess risk (*ER*) with a 95% confidence interval (*CI*) for every 10 μg/m^3^ increase in PM_2.5_ concentration or for every interquartile range (*IQR*) increase in the ratio of the monthly mean PM_2.5_-bound metal concentration to daily PM_2.5_ concentration.

All analyses were conducted using the *mgcv* package in R (version 4.0.2) software. *P* < 0.05 was considered statistically significant.

## Results

Table 2 shows the means and standard deviations of PM_2.5_ concentrations, PM_2.5_-bound metal concentrations, and pediatric outpatient visits for respiratory disease from 2017 to 2019. During the study period, there were 1,707,346 pediatric outpatient visits for respiratory diseases, ranging from 176 to 3,046 visits per day. During the 265-day sampling period, the daily mean concentrations of PM_2.5_, Cd, As, Cr, and Hg were 53.39 μg/m^3^, 1.22, 6.05, 3.22, and 0.06 ng/m^3^, respectively. Based on the secondary and primary concentration limits in GB 3085-2012 and stipulated by the World Health Organization, 19.60%, 69.00%, and 89.10% of the days exceeded the PM_2.5_ concentration limit (75.00, 35.00, and 25.00 μg/m^3^), and 1.10%, 35.80%, and 100% of the days exceeded the Cd, As, and Cr concentration limits, respectively. Cr(VI) accounted for 16.66% of the total Cr concentration (26).

**Table 2:** Daily means and standard deviations of PM_2.5_ concentration, PM_2.5_-bound metal concentrations, and pediatric respiratory outpatient visits from 2017 to 2019.

**Item**	**Units**	**$\bar{x}\pm$s**	**Min**	**P25**	**P50**	**P75**	**Max**
PM_2.5_	(μg/m^3^)	53.39 ± 30.10	6.30	32.45	46.00	65.75	199.00
Ag	(ng/m^3^)	0.22 ± 0.17	0.03	0.10	0.18	0.28	1.45
Al	(ng/m^3^)	114.13 ± 70.66	19.98	66.60	93.70	142.00	495.00
As	(ng/m^3^)	6.05 ± 5.35	1.01	2.68	4.56	7.40	42.70
Ba	(ng/m^3^)	17.66 ± 16.63	4.69	10.00	13.50	19.40	171.19
Be	(ng/m^3^)	0.03 ± 0	0.03	0.03	0.03	0.03	0.03
Bi	(ng/m^3^)	1.64 ± 1.56	0.17	0.62	1.35	2.11	13.20
Cd	(ng/m^3^)	1.22 ± 1.07	0.21	0.52	0.95	1.58	8.30
Co	(ng/m^3^)	0.26 ± 0.14	0.06	0.16	0.23	0.34	0.81
Cr	(ng/m^3^)	3.22 ± 4.83	0.35	1.51	2.47	3.63	70.70
Cu	(ng/m^3^)	21.02 ± 13.04	4.89	11.40	17.00	26.30	85.70
Fe	(ng/m^3^)	396.40 ± 318.27	130.69	243.00	319.00	461.00	4,420.00
Hg	(ng/m^3^)	0.06 ± 0.09	0.00	0.02	0.05	0.08	0.90
Li	(ng/m^3^)	0.65 ± 0.48	0.03	0.33	0.54	0.82	3.36
Mn	(ng/m^3^)	25.24 ± 14.21	7.17	14.70	21.30	30.70	91.40
Mo	(ng/m^3^)	0.76 ± 0.43	0.11	0.46	0.65	0.97	2.65
Ni	(ng/m^3^)	2.47 ± 2.11	0.06	1.00	1.91	3.22	14.60
Pb	(ng/m^3^)	32.21 ± 25.77	5.19	12.40	25.00	41.60	155.00
Sb	(ng/m^3^)	3.49 ± 2.45	0.81	1.70	2.88	4.31	16.25
Se	(ng/m^3^)	2.54 ± 1.59	0.43	1.38	2.10	3.27	9.25
Sn	(ng/m^3^)	5.21 ± 3.95	1.19	2.80	4.13	6.51	31.40
Sr	(ng/m^3^)	2.57 ± 3.93	0.33	1.31	1.84	2.77	42.76
Th	(ng/m^3^)	0.03 ± 0.00	0.03	0.03	0.03	0.03	0.03
Tl	(ng/m^3^)	0.29 ± 0.24	0.04	0.13	0.23	0.36	1.37
U	(ng/m^3^)	0.04 ± 0.03	0.02	0.02	0.04	0.05	0.14
V	(ng/m^3^)	4.55 ± 5.05	0.12	1.00	3.14	5.67	34.00
Zn	(ng/m^3^)	139.15 ± 90.75	36.00	81.80	108.00	173.00	736.00
Respiratory diseases outpatient visits	*n*	1,571 ± 455	176	1,284	1,546	1,805	3,046
AURIs	*n*	860 ± 262	102	665	850	1,048	1,559
FLU&PN	*n*	67 ± 52	2	30	51	94	404
ALRIs	*n*	297 ± 128	37	185	296	401	649

$\bar{x}$, mean; s, standard deviation; P25, lower quartile; P50, median; P75, upper quartile; Max, maximum value; Min, minimum value; AURIs, acute upper respiratory infections; FLU&PN, influenza and pneumonia; ALRIs, acute lower respiratory infections.

**Figure 1:**
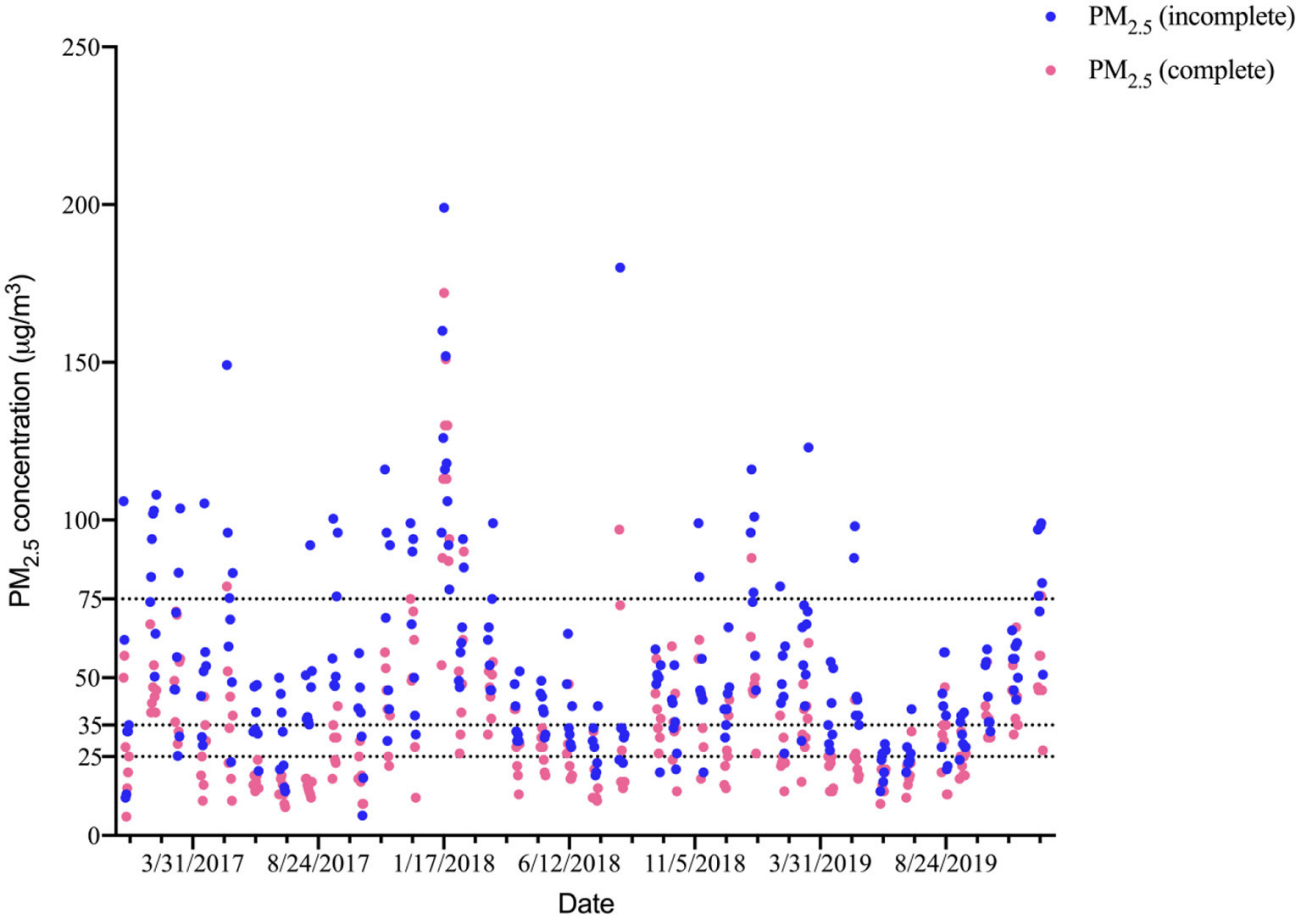
Trends in the complete and incomplete PM_2.5_ concentration (μg/m^3^) on the sampling dates in Yuexiu district and a comparison with World Health Organization and GB 3085-2012 standards. The three lines represent PM_2.5_ concentration limits in GB 3085-2012 (75.00 and 35.00 μg/m^3^) and World Health Organization (25.00 μg/m^3^).

As shown in Figure 1, the PM_2.5_ concentrations were higher in winter and lower in summer. The time series analysis required PM_2.5_ concentration information on consecutive dates; however, there were incomplete data for the 24-h mean PM_2.5_ concentrations for the 26 metals (data from only 265 days were available). However, the trends in the PM_2.5_ concentrations on these 265 days and the trends in the PM_2.5_ concentrations over the entire study period were similar. Therefore, we used the continuously monitored PM_2.5_ data to fit the incomplete PM_2.5_ concentration data by correlation and linear regression, and the results showed a strong correlation (*r* = 0.74, *P* < 0.01) and were significant (β = 0.97, *P* < 0.05).

### Source classification

**Figure 2:**
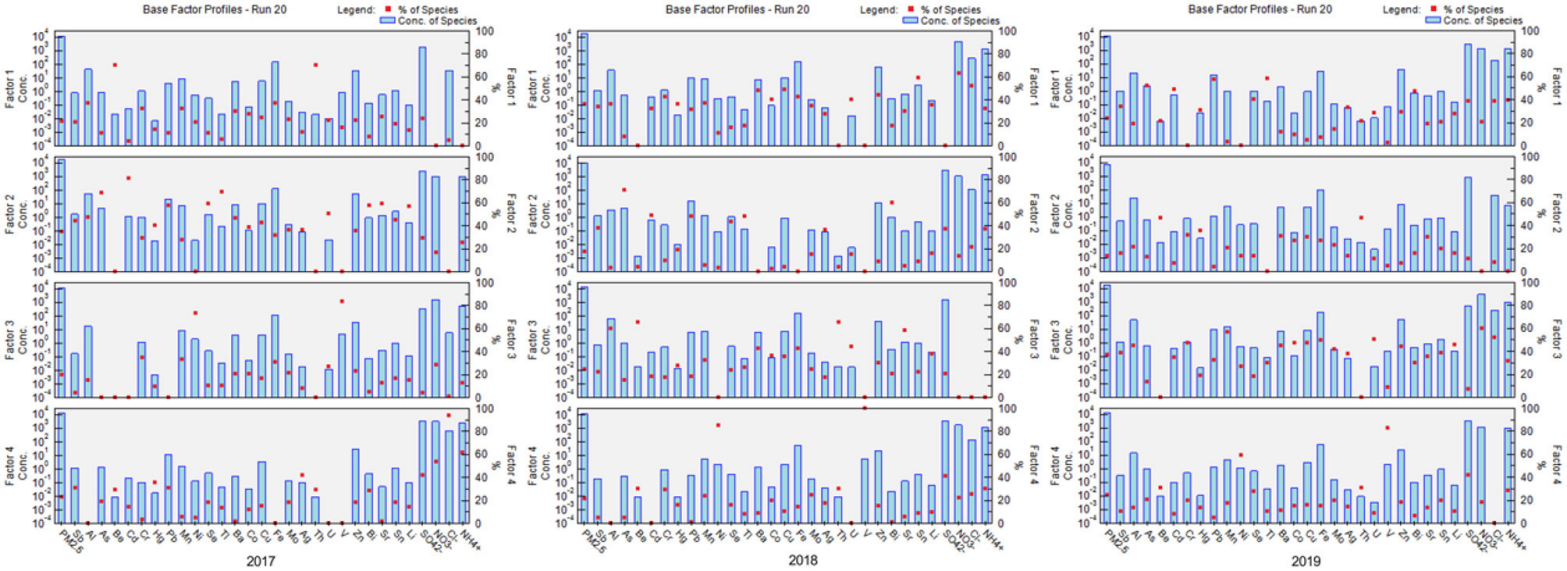
Positive matrix factorization (PMF) analysis of the distribution of sources of PM_2.5_-bound metal from 2017 to 2019. Red dot means percentage ratio of each kind of species, blue bar means concentration of each species.

We used PMF to analyze the sources of PM_2.5_-bound metals. First, Cattle's scree test was performed to determine the number of principal components. Next, based on the source spectrum database in China (15, 27, 28), EPA PMF 5.0 was employed to quantify PM_2.5_-bound metal sources. Based on the Kaiser criterion in the Cattle's scree test, we retained four principal factors as pollution sources. Figure 2 presents the PMF results from 2017 to 2019. These results reveal that the six potential sources chosen by the model were street dust, motor vehicles, ships and heavy oil, secondary nitrates, boiler combustion, and aerospace manufacturing.

Among these six potential sources, street dust was characterized by high concentrations of Al, Cr, and Fe; motor vehicles were characterized by high concentrations of Pb and Zn; ships and heavy oil were characterized by high concentrations of Ni and V; secondary nitrates were characterized by high concentrations of sulfate (${\text{SO}}_{4}^{2-}$), nitrate (${\text{NO}}_{3}^{-}$), and ammonium (${\text{NH}}_{4}^{+}$); boiler combustion was characterized by high concentrations of As; and aerospace manufacturing was characterized by high concentrations of Be.

**Figure 3:**
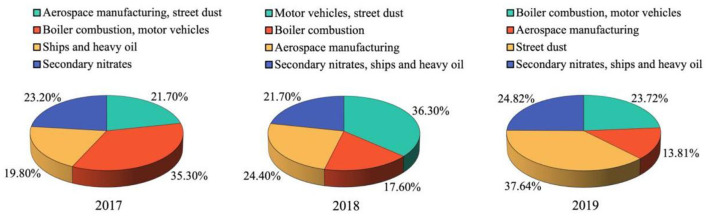
Source contributions to PM_2.5_-bound metals from 2017 to 2019.

The source contributions to PM_2.5_-bound metals from 2017 to 2019 are shown in Figure 3. The highest contributions in the 3 years were from motor vehicles and boiler combustion (2017), motor vehicles and street dust (2018), and street dust (2019).

### Human health risk assessment of exposure to PM_2.5_-bound metals

Table 1 shows the exposure parameters and non-carcinogenic risk assessment results for exposure to PM_2.5_-bound Al, As, Ba, Cd, Co, Cr(VI), Mn, Ni, Se, and V, and the CRs for exposure to PM_2.5_-bound As, Cd, Co, Cr(VI), Ni, and Pb. Only Mn posed a non-carcinogenic health risk, and thus the other PM_2.5_-bound metals were not included in the time series analysis. The mean CRs of As, Cd, Co, Cr(VI), Ni, and Pb exceeded the acceptable risk level (1 × 10^−6^); therefore, the relatively high exposures of children to these PM_2.5_-bound metals are of concern.

### Associations between concentrations of PM_2.5_ and its metal constituents and pediatric respiratory outpatient visits

**Figure 4:**
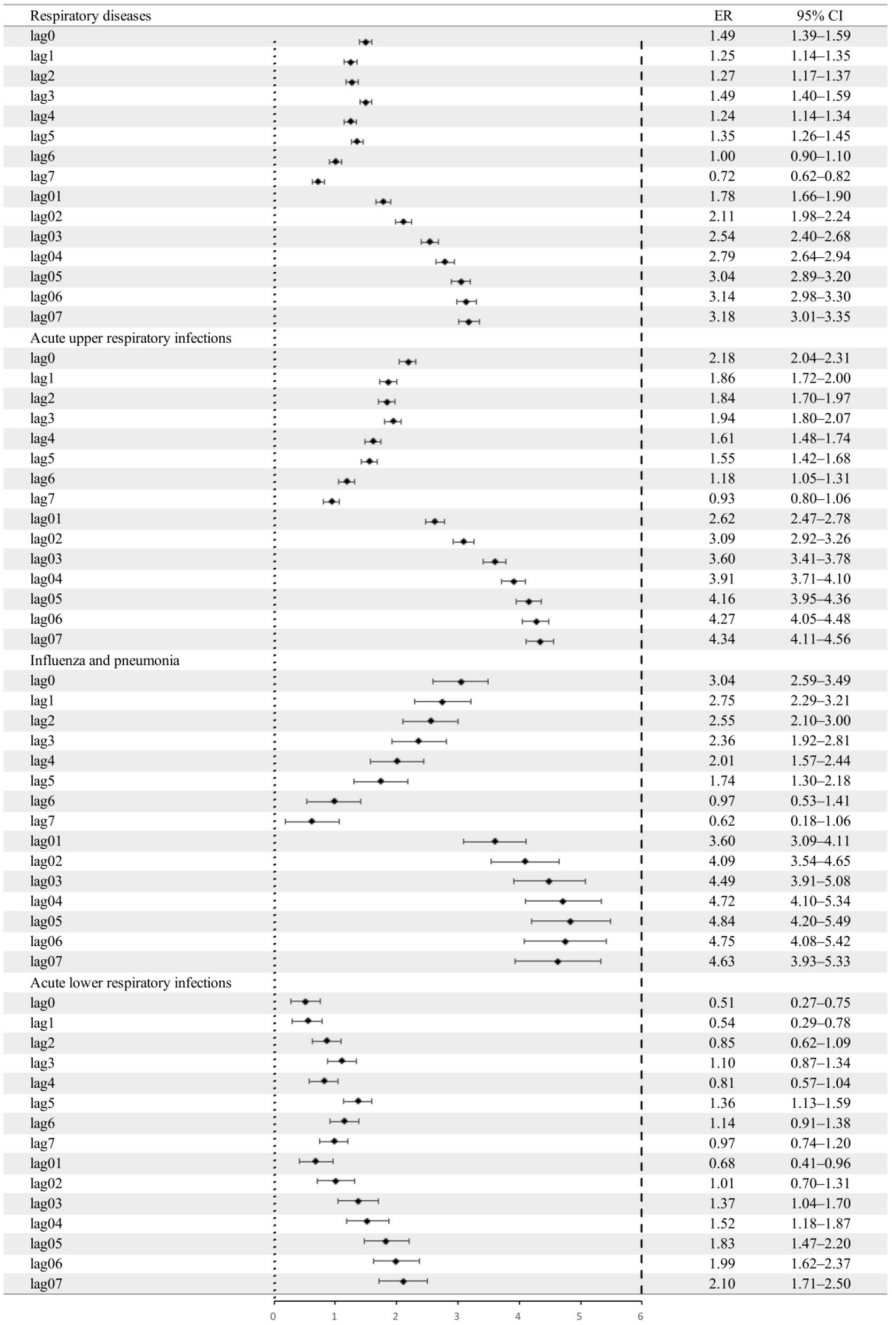
Excess risk and 95% *CI*s for the percentage increase in outpatient visits for respiratory diseases per 10 μg/m^3^ increase in the concentration of PM_2.5_. *CI*, confidence interval, the black dot indicates the mean excess risk, the black vertical line corresponds to 95%*CI*, and the vertical dotted line at 0 represents the reference value.

Figure 4 shows the PM_2.5_-associated *ER* for total pediatric outpatient visits for diseases and pediatric outpatient visits for three respiratory diseases. The lag structure of changes in pediatric outpatient visits for respiratory diseases was associated with a 10 μg/m^3^ increase in PM_2.5_ daily concentration. PM_2.5_ concentration significantly affected the number of daily pediatric outpatient visits due to AURIs, FLU&PN, and ALRIs.

Regarding the lag effect, lag3 was specifically associated with a 1.49% (95% *CI*: 1.39–1.59%) increase in daily pediatric outpatient visits for respiratory diseases per 10 μg/m^3^ increase in PM_2.5_ concentrations. Regarding pediatric outpatient visits for the three causes of respiratory diseases, the highest lag effects were a 3.04% (95% *CI*: 2.59–3.49%) increase in FLU&PN at lag0, a 2.18% (95% *CI*: 2.04–2.31%) increase in AURIs at lag0, and a 1.36% (95% *CI*: 1.13–1.59%) increase in ALRIs at lag5.

In terms of the cumulative lag effect, the strongest effect was at lag07 for all but FLU&PN—the FLU&PN daily outpatient visits reached a peak at lag05. In terms of total pediatric outpatient visits for respiratory diseases, each 10 μg/m^3^ increase in the concentration of PM_2.5_ was associated with a 3.18% (95% *CI*: 3.01–3.35%) increase in visits. FLU&PN visits were most affected, with an increase of 4.84% (95% *CI*: 4.20–5.49%), which was consistent with the results of the lagged effects model.

### Relationships between PM_2.5_-bound metal concentrations and respiratory diseases in children

We included As, Cd, Co, Cr(VI), Mn, Ni, and Pb in the GAM because these metals were found in the risk assessment to have carcinogenic and non-carcinogenic risks in children. Next, as mentioned, the ratio of the monthly mean concentration of PM_2.5_-bound metals to the daily PM_2.5_ concentration was calculated to compensate for the absence of daily metal information. The *ER* for respiratory disease outpatient visits is given per *IQR* increase in the monthly mean ratio of metal concentration to PM_2.5_ concentration.

**Figure 5:**
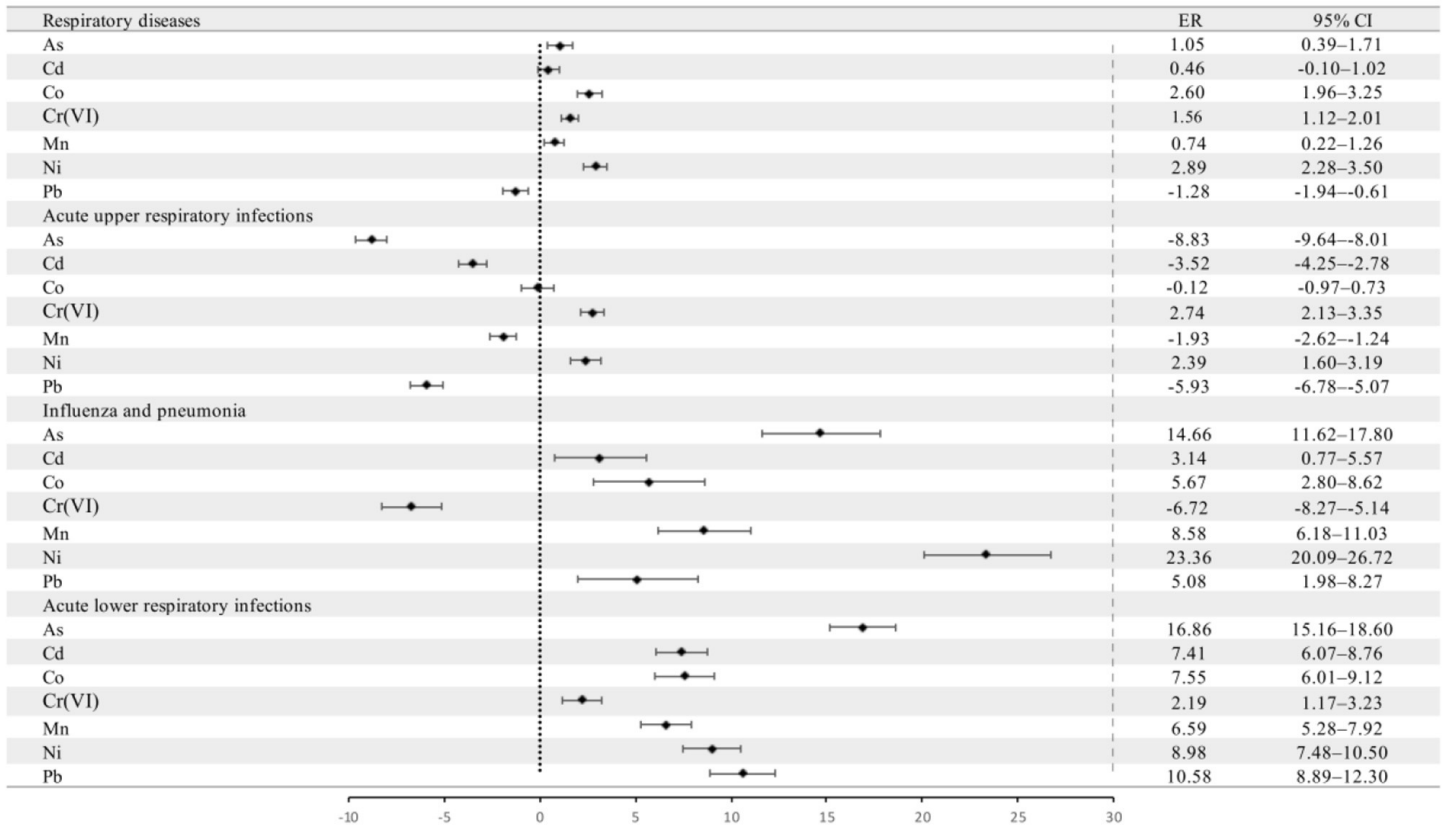
Percentage change (*ER* and 95% *CI*) in pediatric outpatient visits for various respiratory diseases per *IQR* increase in the concentrations of As, Cd, Co, Cr(VI), Mn, Ni, and Pb in PM_2.5_ at time lag0. *CI*, confidence interval, black dot indicates the mean excess risk, the black vertical line corresponds to 95%*CI*, and the vertical dotted line at 0 represents the reference value.

Figure 5 shows the changes in total pediatric outpatient visits for respiratory diseases and pediatric outpatient visits for the three respiratory diseases associated with an *IQR* increase in the monthly mean metal-to-PM_2.5_ concentration ratio. The increase in monthly mean Ni concentration/PM_2.5_ concentration had the greatest effect, increasing total pediatric outpatient visits for respiratory diseases by 2.89% (95% *CI*: 2.28–3.50%) and for daily pediatric outpatient visits for FLU&PN by 23.36% (95% *CI*: 20.09–26.72%). An *IQR* increase in the monthly mean Cr(VI) and As concentrations/PM_2.5_ concentration had the greatest effects on daily pediatric outpatient visits for AURIs and ALRIs, respectively.

**Figure 6:**
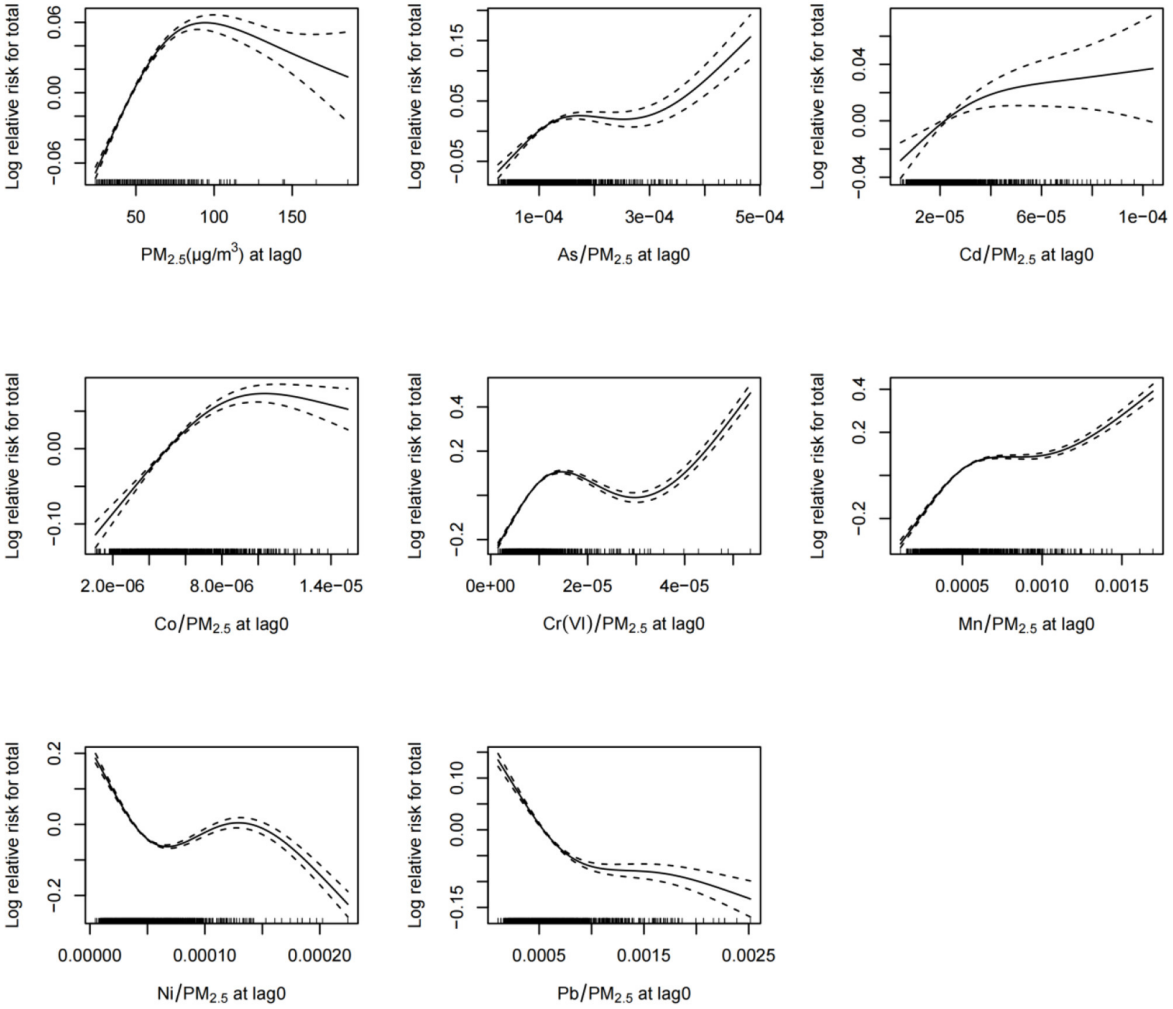
Exposure–response relationships between the concentrations of ambient PM_2.5_ and PM_2.5_-bound metals and total pediatric outpatient visits for respiratory diseases at lag0. *CI*, confidence interval, dotted line represents confidence interval; solid line represents log relative risk.

The exposure–response relationship curves in Figure 6 indicate that the daily concentrations of PM_2.5_ and PM_2.5_-bound metals were associated with total daily pediatric outpatient visits for respiratory diseases at lag0 (Figure 6). The relationships between exposure to PM_2.5_, As, Cd, Co, Cr(VI), Mn, and total daily pediatric outpatient visits for respiratory diseases show an “N” shape, suggesting that lower concentrations of PM_2.5_ and these six metals increased the total daily pediatric outpatient visits for respiratory diseases. The exposure–response curve for Ni shows a “~” trend, indicating that as Ni exposure increased, the total daily pediatric outpatient visits for respiratory diseases initially decreased, then rose, and then finally decreased.

## Discussion

This study was conducted in three stages. In the first stage, we investigated the pollution characteristics of PM_2.5_-bound metals through PMF source analysis. In the second stage, we identified metals that pose a potential carcinogenic risk and non-carcinogenic risk for children. In the third stage, we used GAM to analyze the associations between ambient PM_2.5_ and PM_2.5_-bound metals and the risk of daily pediatric outpatient visits for respiratory diseases in Yuexiu district, Guangzhou, China, from 2017 to 2019. During the study period, the daily mean PM_2.5_ concentration was 53.39 μg/m^3^ in Yuexiu district; by comparison, a similar study found a concentration of 50.30 μg/m^3^ in Panyu district, Guangzhou (29). Yuexiu district has poor air quality and severe ambient pollution. The PM_2.5_ concentration is typically higher in spring and winter and lower in summer and autumn; these changes might be related to the decrease in temperature and relative humidity in spring and winter, in addition to firecracker burning that takes place during the Spring Festival in China (30).

The PMF source analysis results showed that the primary sources of PM_2.5_-bound metals were motor vehicles and present in street dust; secondary sources were boiler combustion and ships and heavy oil. These results are consistent with previous studies that found that PM_2.5_-bound metals originated mostly from vehicle exhaust and boiler combustion in Zhuhai (28) and Guangzhou (27). According to the source spectrum database in China (15, 27, 28), Al, Cr, and Fe are mainly found in street dust, Pb and Zn are mainly emitted by motor vehicles, Ni and V are mainly produced by ship fuel and heavy oil combustion, and As is mainly produced by boiler combustion.

The health risk assessment outcomes showed that PM_2.5_-bound As, Cd, Co, Cr(VI), Mn, Ni, and Pb posed a risk to children. Only Mn had a non-carcinogenic risk, a finding that is consistent with a similar study (9). In the carcinogenic risk assessment, As, Cd, Co, Cr(VI), Ni, and Pb were found to be potentially carcinogenic in children, with a *CR* order of Cr(VI) > As > Co > Cd > Ni > Pb. This is consistent with the results of a previous study in Yuexiu district (As > Cd) (9). At present, the IARC classifies As, Cd, and Cr(VI) as “I” (Group 1 carcinogens) and Ni as “IIB” (Group 2B carcinogens); hence, more attention should be given to the health effects of As, Cd, Co, Cr(VI), Mn, Ni, and Pb exposure.

We found significant associations between PM_2.5_ concentrations and pediatric outpatient visits for various respiratory diseases. We also found that the effect of PM_2.5_ concentrations on pediatric outpatient visits for various respiratory diseases was cumulative. Regarding the lag effect, we observed that the PM_2.5_ concentration had a significant positive effect on total pediatric outpatient visits for respiratory diseases at lag3 (1.49%; 95% *CI*: 1.39–1.59%). This result is similar to that in a previous study in the same city. However, it is a stronger association than has been found in a different city (25, 31). Regarding the cumulative lag effect, the effect of PM_2.5_ concentration on pediatric outpatient visits for various respiratory diseases increased with the number of cumulative lag days. The most significant effect was for lag07, where each 10 μg/m^3^ increase in PM_2.5_ concentration was associated with a 3.18% (95% *CI*: 3.01–3.35%) increase in total daily pediatric outpatient visits for respiratory diseases. This finding is similar to those of previous studies (25, 31).

Regarding pediatric respiratory outpatient visits for AURIs, FLU&PN, and ALRIs, the *ER* was most significant at lag0 for AURIs and FLU&PN. The *ER* increase slowed as cumulative lag days increased. A few studies have examined the relationship between outpatient visits for different respiratory diseases and PM_2.5_, and most have also only found relationships between PM_2.5_ concentrations and AURIs and FLU&PN (25, 32, 33). Therefore, further exploration of the relevant mechanisms and epidemiological evidence is necessary. Our analysis revealed that As, Cd, Co, Cr(VI), Mn, Ni, and Pb concentrations were associated with pediatric outpatient visits for respiratory diseases. In a similar analysis, Valdés et al. (13) found that Ni and Cr concentrations were significantly associated with higher respiratory mortality. In line with our findings, a study in Canada (6) reported associations between respiratory hospitalizations and Sulfur (S) and Cu, and Mn exposure, and a study in Xian (11) found a significant association between Ni exposure and respiratory mortality.

The mechanisms by which inhaled As, Cd, Co, Cr(VI), Mn, Ni, and Pb adversely affect children's respiratory health have been widely studied (34–39). As is known to cause lung cancer by disrupting immune function and inhibiting immune function-related genes, and as exposure is also associated with non-malignant respiratory diseases (e.g., acute respiratory infections and symptoms of decreased lung function) (36). Low levels of Cd exposure can lead to lung function alterations that can cause pulmonary fibrosis, emphysema, and lung tumors, and Cd toxicity increases oxidative stress that depletes protein-bound glutathione and sulfhydryl groups. Although Cd can enter the body through inhalation, the current study shows that high Cd exposure mainly arises through food intake and smoking. This suggests that particular attention should be given to preventing children being exposed to secondhand smoke (35). Chronic inhalation of Co compounds can lead to respiratory tumors, and Co exposure often leads to interstitial lung disease (34). Inhalation is the primary route of exposure to Cr and can lead to lung cancer, chromosomal damage, asthma, cough, acute bronchitis, and pulmonary edema (35). Exposure to low concentrations of Mn can result in adverse health effects on respiratory organs; Mn is also an immunotoxin and is cytotoxic to lung macrophages, thus making children susceptible to small airway bronchial injury (37). Ni exposure can increase respiratory disease morbidity and mortality, and it promotes the expression of inflammatory factors, contributing to airway inflammation (39). Pb exposure downregulates interleukin-13 expression and can increase the risk of asthma-related immunomodulatory abnormalities in preschool children (38).

Children are a population group that is sensitive to air pollution because they are in a period of growth and development. In addition, children have a relatively rapid respiratory rate and are more vulnerable to respiratory diseases such as asthma, cough, and respiratory inflammation. Studies have shown that respiratory diseases have become the most frequent cause of childhood outpatient visits for illness, and these diseases are a primary medical problem adversely affecting the health and growth of children (40).

Thus, the government should strengthen the supervision and control of three sources of pollution—waste incineration, metal smelting, and traffic—in addition to improving purification measures and rationalizing the separation of traffic flow to relieve traffic pressure. In addition, parents can lower the risk of respiratory diseases in their children by reducing their children's exposure to PM_2.5_, As, Cd, Co, Cr(VI), Mn, Ni, and Pb. This can be achieved by parents not bringing their children to certain areas and not exposing them to secondhand smoke. Finally, parents should also ensure that their children have adequate physical exercise to strengthen their immunity.

This study has several strengths. First, to the extent of our knowledge, it is the first study to use a comprehensive and systematic approach to quantify the associations between exposure to PM_2.5_-bound metals and pediatric outpatient visits for respiratory disease. Second, it examined the total pediatric outpatient visits for respiratory diseases, AURIs, FLU&PN, and ALRIs, while previous studies have investigated only some of these conditions. Third, to obtain reliable results, it used a quasi-Poisson GAM with adjustments for lag effects, DOW, temperature, and humidity.

The limitations of our study should also be noted. First, we included clinical data from only one hospital, which cannot accurately represent all of the pediatric outpatient visits in Yuexiu district. Second, the air pollution exposure concentration we used was obtained from regional air pollution monitoring data, and thus there may have been some errors in the exposure measurement. Third, a certain amount of error was introduced by compensating for the lack of daily data on metal concentrations by calculating the monthly mean metal concentration to daily PM_2.5_ concentration ratio. Few studies have been conducted in this way; therefore, precise comparisons with previous studies were not possible. Nevertheless, relative changes were examined.

## Conclusion

Our findings suggest that PM_2.5_ and PM_2.5_-bound As, Cd, Co, Cr(VI), Ni, and Pb pose potential health risks to children in Guangzhou, China, and can contribute to respiratory disease in this population. Government departments can lower children's risk of respiratory diseases by introducing measures that decrease the production of these pollutants by motor vehicles and reduce the amount of street dust.

## Data availability statement

The original contributions presented in the study are included in the article/supplementary material, further inquiries can be directed to the corresponding author.

## Author contributions

QY designed the study and supervised the research, including funding, text review, and overall quality assurance and control. YZ and SC were helped with the formulation of research methods, software analysis and interpretation of the results, and wrote the original draft of the text. YC and JL were helped with the investigation and review of the data and assisted in the preparation of the original draft of the text. BX and TS were assisted in the implementation of research, data management and investigation, and supervision. All authors contributed to the article and approved the submitted version.
